# *Bothrops jararaca* Snake Venom Inflammation Induced in Human Whole Blood: Role of the Complement System

**DOI:** 10.3389/fimmu.2022.885223

**Published:** 2022-06-02

**Authors:** Thyago Bispo Leonel, Joel José Megale Gabrili, Carla Cristina Squaiella-Baptistão, Trent M. Woodruff, John D. Lambris, Denise V. Tambourgi

**Affiliations:** ^1^ Immunochemistry Laboratory, Instituto Butantan, São Paulo, Brazil; ^2^ School of Biomedical Sciences, Faculty of Medicine, The University of Queensland, St Lucia, QLD, Australia; ^3^ Department of Pathology and Laboratory Medicine, Perelman School of Medicine, University of Pennsylvania, Philadelphia, PA, United States

**Keywords:** human whole blood, inflammation, complement system and inhibitors, *Bothrops jararaca*, snake venom

## Abstract

The clinical manifestations of envenomation by *Bothrops* species are complex and characterized by prominent local effects that can progress to tissue loss, physical disability, or amputation. Systemic signs can also occur, such as hemorrhage, coagulopathy, shock, and acute kidney failure. The rapid development of local clinical manifestations is accompanied by the presence of mediators of the inflammatory process originating from tissues damaged by the bothropic venom. Considering the important role that the complement system plays in the inflammatory response, in this study, we analyzed the action of *Bothrops jararaca* snake venom on the complement system and cell surface receptors involved in innate immunity using an *ex vivo* human whole blood model. *B. jararaca* venom was able to induce activation of the complement system in the human whole blood model and promoted a significant increase in the production of anaphylatoxins C3a/C3a-desArg, C4a/C4a-desArg, C5a/C5a-desArg and sTCC. In leukocytes, the venom of *B. jararaca* reduced the expression of CD11b, CD14 and C5aR1. Inhibition of the C3 component by Cp40, an inhibitor of C3, resulted in a reduction of C3a/C3a-desArg, C5a/C5a-desArg and sTCC to basal levels in samples stimulated with the venom. Exposure to *B. jararaca* venom induced the production of inflammatory cytokines and chemokines such as TNF-α, IL-8/CXCL8, MCP-1/CCL2 and MIG/CXCL9 in the human whole blood model. Treatment with Cp40 promoted a significant reduction in the production of TNF-α, IL-8/CXCL8 and MCP-1/CCL2. C5aR1 inhibition with PMX205 also promoted a reduction of TNF-α and IL-8/CXCL8 to basal levels in the samples stimulated with venom. In conclusion, the data presented here suggest that the activation of the complement system promoted by the venom of the snake *B. jararaca* in the human whole blood model significantly contributes to the inflammatory process. The control of several inflammatory parameters using Cp40, an inhibitor of the C3 component, and PMX205, a C5aR1 antagonist, indicates that complement inhibition may represent a potential therapeutic tool in *B. jararaca* envenoming.

## Introduction

Ophidic accidents are an important public health challenge in several regions worldwide (1). It is estimated that approximately 1.8 to 2.7 million envenomings occur annually, resulting in between 81,000 and 138,000 deaths and approximately three times more amputations and other permanent disabilities each year (2, 3).

Due to the remarkable social and economic impact of such incidents, after the 10th meeting of the Strategic and Technical Advisory Group on Neglected Tropical Diseases (Geneva, March 2017), envenomations by snakes were included on June 2017 in the category of priority neglected tropical diseases of the World Health Organization (WHO), which currently includes 19 other diseases (4).

In 2019, new guidelines for the prevention and control of snake accidents were developed by the World Health Organization to reduce mortality and disability by 50% before 2030. To accomplish this task, four objectives were outlined: (*i*) empower and involve communities; (*ii*) ensure safe and effective treatments; (*iii*) strengthen health systems; and (*iv*) increase partnerships, coordination, and collaborative resources (5).

Among the various snake families, only Elapidae, Hydrophiidae, Viperidae, Crotalidae and Colubridae have venomous species (6). The genus *Bothrops* (family Viperidae) includes more than 30 species and subspecies that are widely distributed in the neotropical region, from southern Mexico to northern Argentina and on some Caribbean islands (7, 8).

In Brazil*, Bothrops jararaca* Wied, 1824 (*B. jararaca*) represents a species with a greater severity of accidents among humans. The clinical manifestations of the envenoming caused by *B. jararaca* snakes are complex and characterized by prominent local effects, including pain, edema, ecchymosis, blisters, abscesses, and necrosis, which may progress to tissue loss, physical disability, or amputation. Systemic signs may also occur, such as hemorrhage (gingival bleeding, hematuria, and epistaxis), coagulopathy, shock, and acute renal failure (9–11).

The rapid development of *Bothrops* envenoming manifestations is accompanied by the presence of mediators of the inflammatory process (12, 13). Antivenom has specificity for the main toxins present in the venom and thus is able to neutralize them in circulation; however, it does not exert the same effect on the inflammatory mediators (9). Regardless of the cause, inflammation always seeks to restore homeostasis by establishing the stages of healing and reconstitution of damaged tissue (14). The mechanisms occurring in the inflammatory process include the activation of the complement system.

The complement system is composed of more than 40 plasma and cell surface proteins that interact with each other and other molecules, thereby generating enzyme complexes with proteolytic activity that activate, amplify, and regulate important functions in the immune and inflammatory response (15). Its activation can be initiated by three main pathways, *i.e.*, the alternative pathway (AP), classical pathway (CP) and lectin pathway (LP) (16, 17). All three pathways result in the formation of C3-convertase (C3bBb in AP and C4bC2a in CP and LP), cleavage of component C3 with generation of opsonin C3b and anaphylatoxin C3a. C3b is involved in the formation of the C5-convertase (C3bBbC3b in AP and C4b2a3b in CP and LP), which in turn cleaves C5, thereby generating C5b and the anaphylatoxin C5a. In the terminal stage, C5b interacts with C6, C7, C8 and several C9 molecules to form the membrane attack complex (C5b-9n or MAC), which generates hydrophilic pores and induces cell lysis (17).

The anaphylatoxins C3a, C4a and C5a are biologically active fragments that are constantly released during the activation of the complement system. These small peptides (10-14 kDa) are potent inflammatory mediators that exert their effects through interactions with specific receptors in various cell types. The interaction of anaphylatoxins with their respective receptors, C3aR, C5aR1 and C5aR2, present in leukocytes and vascular endothelial cells triggers important events in the conduction of the inflammatory response, including recruitment of immune cells to the site of injury, induction of oxidative explosion and promotion of vascular permeability (18–20).

In a previous study conducted with samples of venoms from 19 species of snakes of the genus *Bothrops* present in Brazil, we showed that all venoms were able to activate in the human serum the classical pathway of the complement system in the absence of sensitizing antibodies. Some of these venoms activated other pathways of the system, *i.e.*, AP and LP. The activity of metalloproteases and serinoproteases was fundamental in the generation of large amounts of the anaphylatoxins C3a, C4a and C5a. In addition to the activation of the cascade, the direct cleavage of C3 and C4 or inactivation of the C1-INH regulator contributed to this event. Metalloprotease and serinoprotease inhibitors prevented the cleavage of C3 and C4 by the action of venoms, thereby confirming the action of these enzymes on the complement system (21, 22).

Among the complement components, C3 and C5 have been considered important molecular targets to be neutralized due to their biological contribution in tissue and cellular damage mediated by the complement. Currently, there are a variety of molecules capable of inhibiting C3, C5, C5a and its receptors (C5aR) (23–25).

Among these new antagonists, Cp40, an analog of the peptide inhibitor of C3, compstatin, showed strong efficacy in several models of human diseases, such as sepsis (26), hemorrhagic shock (27), periodontal disease (28, 29), nocturnal paroxysmal hemoglobinuria (30), hemodialysis-induced inflammation (31), glomerulopathy C3 (C3G) (24), acute respiratory distress syndrome (ARDS) of COVID-19 (32), and cobra envenomation (33). In addition, compstatin derivatives are currently under clinical development and evaluation for the treatment of various diseases (27).

PMX53, a cyclic hexapeptide that is active orally and metabolically stable, has shown efficacy in the treatment of various inflammatory diseases, including arthritis, ischemia and reperfusion injuries, sepsis, inflammatory bowel disease and diseases of the central nervous system (34–38). Studies on changes in the PMX53 phenylalanine residue resulted in the discovery of a new antagonist, PMX205 (hydrocinnmate-[OpdChaWR]), which increased lipophilicity, metabolic stability of the compound, and its potency in an inflammatory bowel disease model (39). In addition, PMX205 presented a higher brain penetration capacity than PMX53, which has enabled its use in several models of neurodegenerative diseases (40–42) Currently, these peptide antagonists are the most widely used inhibitors for the study of C5aR1/CD88 (20).

Experimentally, the human whole blood model has been used to evaluate the interaction between the complement system and Toll receptors (TLRs) (43–45). In this model, lepirudin (refludan), a specific thrombin inhibitor that does not affect complement cascade activity, such as anticoagulant, has allowed for the study of the interaction between coagulation and complement systems (43, 46). Recently, an alternative anticoagulant, the peptide GPRP (Gly-Pro-Arg-Pro), which is able to inhibit the action of thrombin on fibrinogen without impairing complement cascade activity, was developed and successfully used in *ex vivo* human whole blood model (47).

In the present study we aimed to analyze the action of snake venom *B. jararaca* on the complement system and cellular surface receptors involved in innate immunity using the *ex vivo* human whole blood model adapted by Johnson et al. (47) and the modulation of these parameters using complement system-specific inhibitors.

## Materials and Methods

### Venom

The venom of the snake *B. jararaca* was supplied by the Laboratory of Herpetology of the Butantan Institute in lyophilized form and maintained at -20°C. The protein and endotoxin contents were quantified by using a BCA assay protein kit (Pierce) and PYROGENT™ Plus Gel Clot LAL Assay (Lonza, USA), respectively, according to the manufacturers’ recommendations. Endotoxin was present in the venom at a level below the assay’s sensitivity (< 0.125 EU/mL). The protein concentration of the venom samples was adjusted to 5 mg/mL with sterile saline solution, aliquoted and stored at -80°C until use.

### Human Whole Blood Model

The experiments using the human whole blood model were developed according to the protocols established by Mollnes et al. (43) and Brekke et al. (44, 45) and recently adapted by Johnson and collaborators (47). Blood was collected from healthy volunteers in polypropylene tubes containing the GPRP peptide (GenOne Biotechnologies, Brazil; 8 mg *per* mL of blood). For the assay, a total volume of 1 mL was used, with 720 μL of blood, 140 μL of sterile saline solution and 140 μL of sterile saline solution containing increasing concentrations of *B. jararaca* venom. The samples were incubated at 37°C for 30, 60 or 120 minutes under agitation. After incubation, aliquots of 500 μL were collected to analyze the expression of cell markers by flow cytometry. The remaining material was centrifuged at 405 x g and 4°C for 10 minutes to obtain the plasma. After this stage, EDTA was added to the plasma samples (final concentration 10 mM), and they were aliquoted and stored at -80°C.

### Inhibition of the Complement System With Cp40 and PMX205

To assess the role of complement in the inflammatory events promoted by *B. jararaca* venom, human whole blood was pretreated with either the compstatin analog Cp40 (C3/C3b inhibitor, 20 µM) (48) or PMX205 (C5aR1 antagonist, 20 µM) (40) or with the appropriate vehicle as a control, *i.e.*, saline (Cp40) or 5% glucose (PMX205), for 5 minutes at room temperature. Next, the samples were treated with *B. jararaca* venom (50.0 μg/mL) or sterile saline solution (negative control) (14% of the total volume, v/v) for 60 minutes at 37°C. After incubation, the material was centrifuged at 405 x g and 4°C for 10 minutes to obtain the plasma. After this stage, it was added EDTA to the plasma (final concentration 10 mM), and the samples were stored at -80°C.

### Dosage of Anaphylatoxins and sTCC

The presence of C3a/C3a-desArg, C4a/C4a-desArg and C5a/C5a-desArg in the samples of plasma collected, as described above, was evaluated using the BD CBA Human Anaphylatoxin Kit, following the manufacturer’s instructions (BD Biosciences, California, USA). The presence of the soluble complement terminal complex (sTCC, SC5b-9) was evaluated by ELISA in plasma samples from human whole blood assays using the MicroVue SC5b-9 Plus EIA kit following the manufacturer’s instructions (Quidel Corporation, California, USA).

### Quantification of Cytokines and Chemokines

The presence of the cytokines IL-1β, IL-6, IL-10, IL-12p70 and TNF in plasma was determined with the BD Cytometric Bead Array (CBA) Human Inflammatory Cytokines kit based on the manufacturer’s instructions (BD Biosciences, California, USA). The presence of the chemokines IL-8, MCP-1, MIG, RANTES and IP-10 in human plasma was determined with the BD Cytometric Bead Array (CBA) Human Chemokines kit based on the manufacturer’s instructions (BD Biosciences, California, USA).

### Analysis of the Expression of Surface Markers in Leukocytes

The samples were submitted to the treatments described above and analyzed for the expression of the cell surface markers CD11b, CD14, C5aR1, C3aR, TLR2 and TLR4 on the surface of leukocytes labeled with specific antibodies for the monocyte (CD33) and granulocyte (CD66b) populations. After the blood treatment, red blood cells were lysed with BD FACS Lysing Solution buffer (BD Biosciences, California, USA). Subsequently, the cells were centrifuged at 405 g at 4°C for 10 minutes, resuspended and marked with monoclonal antibodies from BD Biosciences (California, USA) or eBioscience (California, USA), which were diluted in a 1:5 ratio with anti-CD11b PE (VIM12 clone), anti-CD14 FITC (clones 61D3 and TüK4) and anti-CD33 APC; in a 1:10 ratio with anti-C5aR FITC (clone 8D6), anti-C3aR PE (clone 17), anti-TLR2 PE (clone TL2.1) and anti-TLR4 PE (clone HTA125); and in a 1:20 ratio with CD3 APC-Cy7, CD19 PE-Cy7 and CD66b Alexa647. Monoclonal mouse IgG1k PE and IgG2ak FITC mice were also used as isotypic controls. After 30 minutes of incubation, 275 μL of FACS buffer was added, and the cells were analyzed in a FACSCanto II flow cytometer (BD Biosciences, California, USA) using SOFTWARE BD FACSDiVa, version 4.1 (BD Bioscience, California, USA). The results were expressed as median fluorescence intensity (MFI), determined from the acquisition of 20,000 events.

### Statistical Analysis

Data were expressed as mean ± standard error and statistically analyzed with GraphPad Prism 9.3.1 software (La Jolla, California, USA). For comparisons, statistical analyses were performed using Student’s t test or one-way ANOVA, followed by Tukey’s multiple comparison test. Differences were considered significant at p ≤ 0.05.

## Results

### *B. jararaca* Venom Activates the Complement System in Human Whole Blood

**Figure 1:**
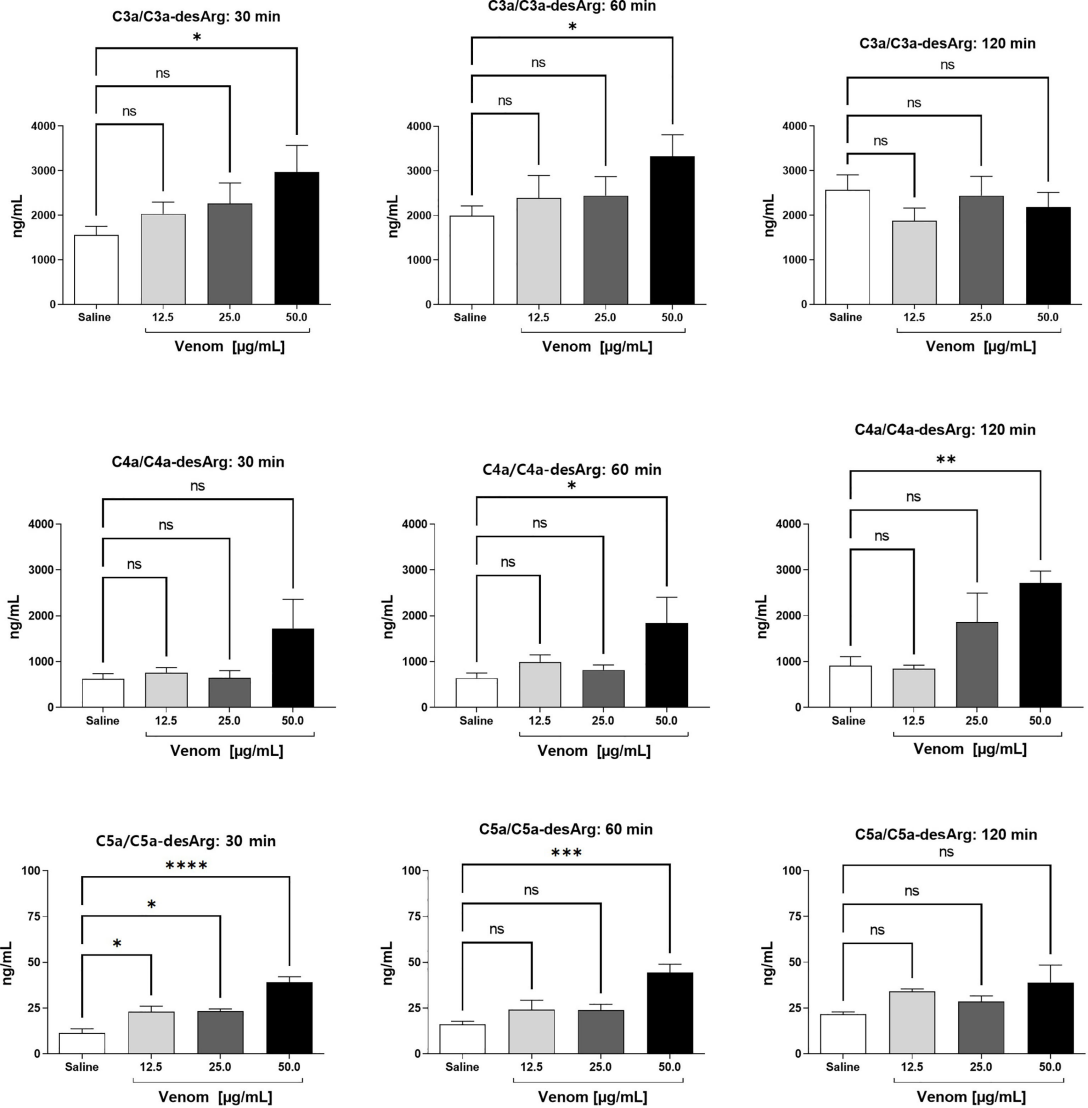
C3a/C3a-desArg, C4a/C4a-desArg and C5a/C5adesArg levels in human plasma after treatment with *B. jararaca* venom. Human blood samples containing the peptide GPRP (8 mg/mL) were incubated with increasing concentrations of *B. jararaca* venom or sterile saline solution for 30, 60 and 120 minutes at 37°C. After plasma collection and dilution, the production of anaphylatoxins C3a/C3a-desArg (1:5000), C4a/C4a-desArg (1:5000) and C5a/C5a-desArg (1:1000) was evaluated by cytometric bead array (CBA). The results are presented as the mean ± SEM from three independent tests. ns, not significant; *p ≤ 0.05, **p ≤ 0.005, ***p ≤ 0.0005, ****p < 0.0001.

**Figure 2:**
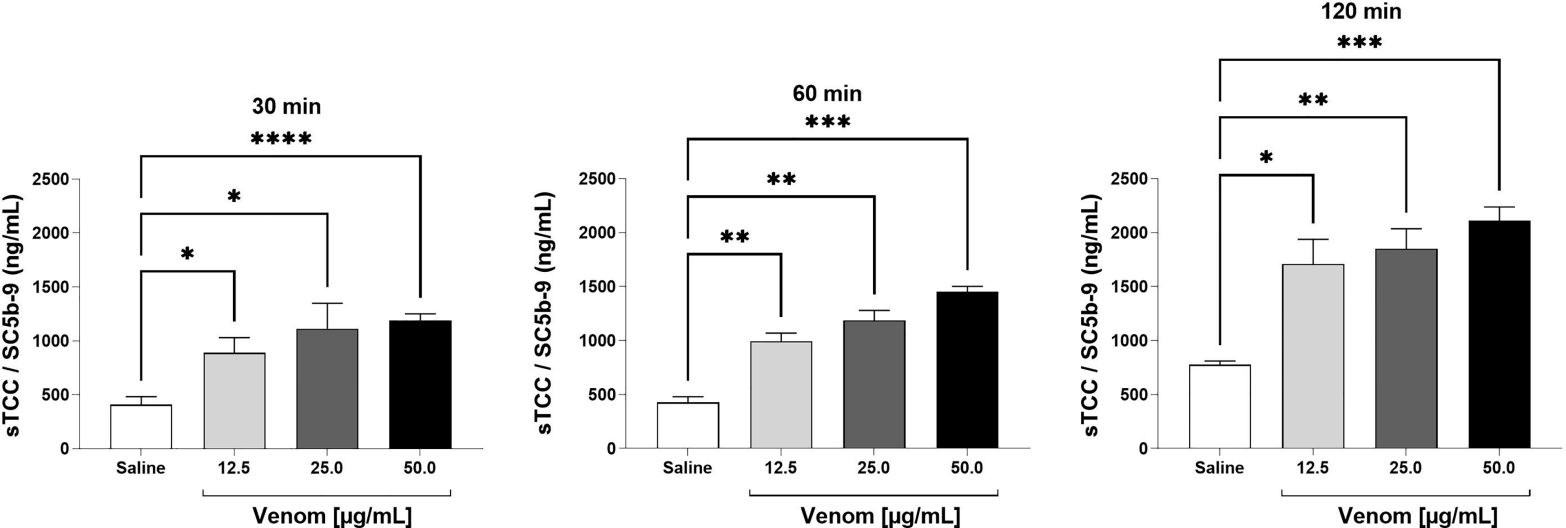
SC5b-9 levels in human plasma after exposure to *B. jararaca* venom. Human blood samples containing the peptide GPRP (8 mg/mL) were incubated with increasing concentrations of *B. jararaca* venom or sterile saline solution for 30, 60 and 120 minutes at 37°C. After plasma collection and dilution (1:40), the presence of the soluble complement terminal complex (sTCC, SC5b-9) was analyzed by ELISA. The results are represented as the mean ± SEM of duplicates from three independent experiments. *p ≤ 0.05, **p ≤ 0.005, ***p ≤ 0.0005, ****p < 0.0001.

Incubation of human blood with increasing concentrations of *B. jararaca* venom resulted in the activation of the complement system as determined by the quantification of the generation of anaphylatoxins (**Figure 1**) and sTCC (**Figure 2**).

A significant increase in the production of C3a/C3a-desArg was detected after 30 and 60 minutes of incubation of human blood with 50.0 μg/mL venom. A venom concentration of 50.0 μg/mL induced a significant increase in C4a/C4a-desArg production at 60 and 120 minutes. *B. jararaca* venom induced a significant increase in C5a/C5a-desArg production when incubated for just a period of 30 minutes at all concentrations. After 60 minutes of incubation, only the venom at a concentration of 50.0 μg/mL induced a significant increase in the production of C5a/C5a-desArg. At 120 minutes, both C3a and C5a production show no difference from the control at all venom concentrations tested (**Figure 1**).

sTCC measurements showed that the venom at a concentration of 12.5 μg/mL induced a significant increase in this macromolecular complex production when incubated for a period of 30 minutes. With 60 and 120 minutes of incubation, the venom was able to induce a significant increase in sTCC production at the three concentrations used (12.5, 25.0 and 50.0 μg/mL) when compared to the negative control (**Figure 2**).

### *B. jararaca* Venom Induces the Production of TNF-α and Chemokines in the Human Blood

**Figure 3:**
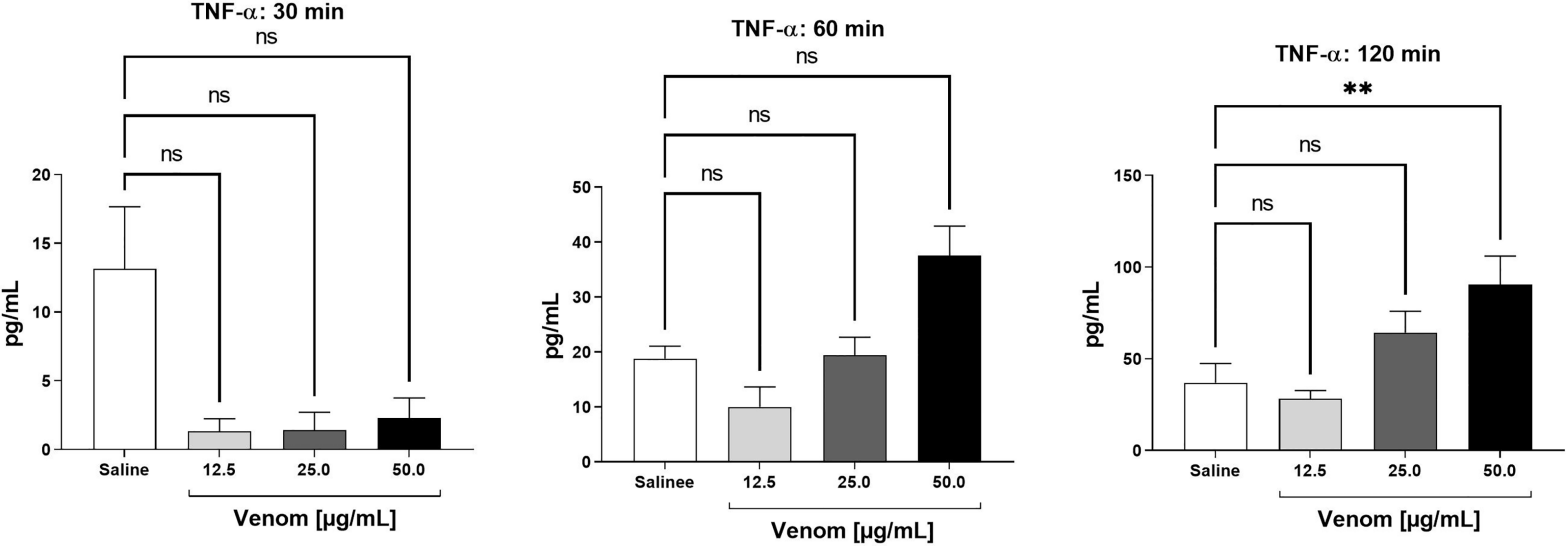
TNF-α levels in human plasma after treatment with *B. jararaca* venom. Human blood samples containing GPRP (8 mg/mL) were incubated with increasing concentrations of *B. jararaca* venom or sterile saline solution for 30, 60 and 120 minutes at 37°C. After plasma collection and dilution (1:2), the presence of TNF-α was evaluated by cytometric bead array (CBA). The results are represented as the mean ± SEM of duplicates from three independent tests. ns, not significant; **p ≤ 0.005.

The presence of the cytokines IL-1β, IL-6, IL-10, IL-12p70 and TNF-α was evaluated in plasma samples obtained from human whole blood assays treated with increasing concentrations of *B*. *jararaca* venom (12.5, 25.0 or 50.0 μg/mL) or in sterile saline solution (negative control) for 30, 60 and 120 minutes at 37°C. **Figure 3** shows time- and dose-dependent curves of the production of TNF-α after incubation of *B. jararaca* venom with human blood. Increased levels of this cytokine compared to the control group were detected after 120 minutes of incubation with 50.0 μg/mL venom. The presence of the other cytokines tested, *i.e.*, IL-1β, IL-6, IL-10, and IL-12p70, was below the detection limit of the assays (data not shown).

**Figure 4:**
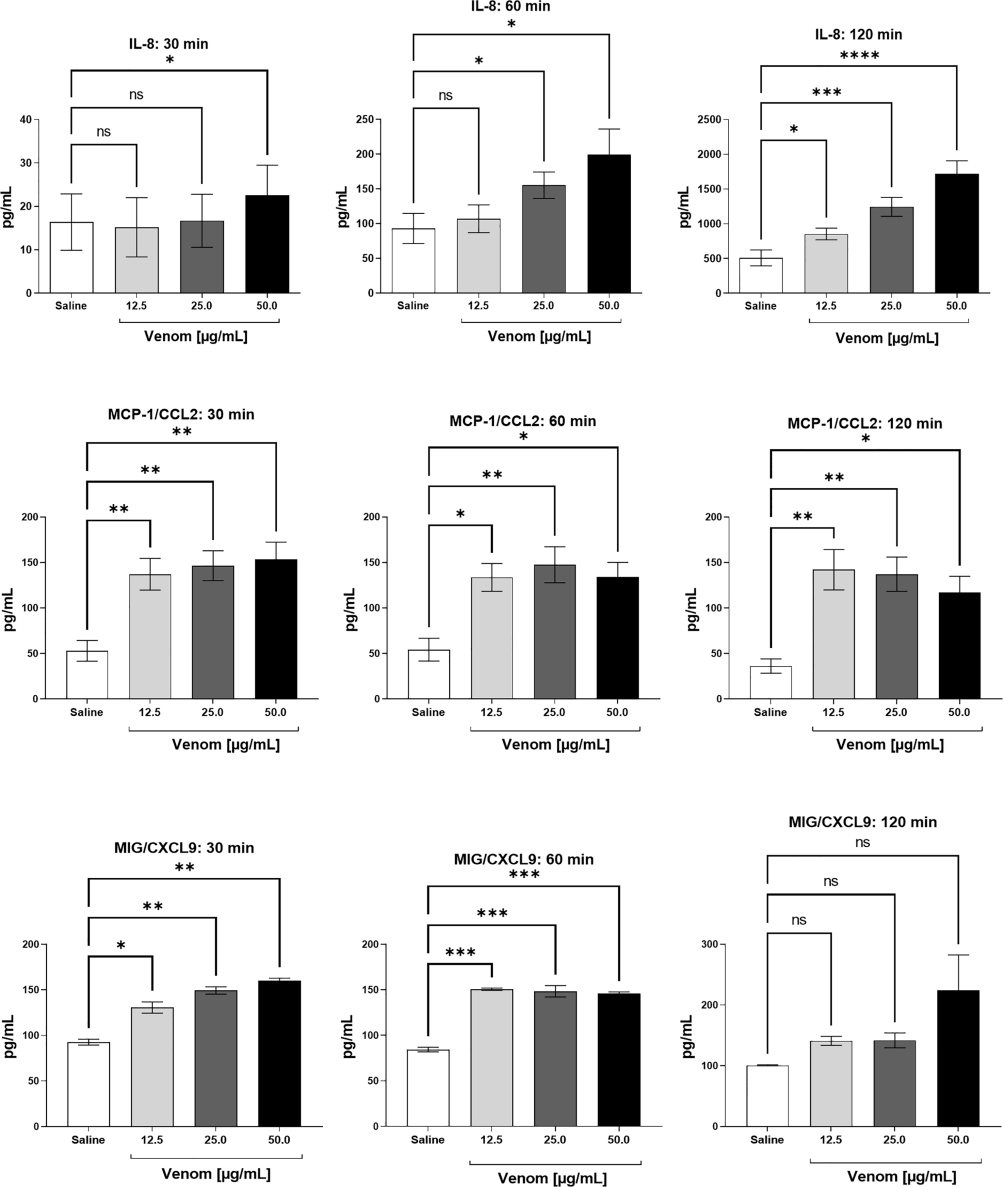
Chemokine levels in human plasma after treatment with *B. jararaca* venom. Human blood samples containing GPRP (8 mg/mL) were incubated with increasing concentrations of *B. jararaca* venom or sterile saline solution for 30, 60 and 120 minutes at 37°C. After plasma collection and dilution (1:2), the presence of chemokines was evaluated by cytometric bead array (CBA). The results are represented as the mean ± SEM of duplicates from three independent tests. ns, not significant; *p ≤ 0.05, **p ≤ 0.005, ***p ≤ 0.0005, ****p < 0.0001.

**Figure 4** shows a time- and dose-dependent increase in IL-8 production, which was detected after 30 minutes of incubation with 50.0 μg/mL of *B. jararaca* venom and increases after 60 and 120 min of incubation. Moreover, the venom induced a high production of MCP-1 at all times and venom concentrations tested; for MIG, it was detected a large amount of this chemokine at 30 and 60 min in all venom concentrations tested.

### *B. jararaca* Venom Induces Blood Leukocyte Cell Surface Molecules Modulation

**Figure 5:**
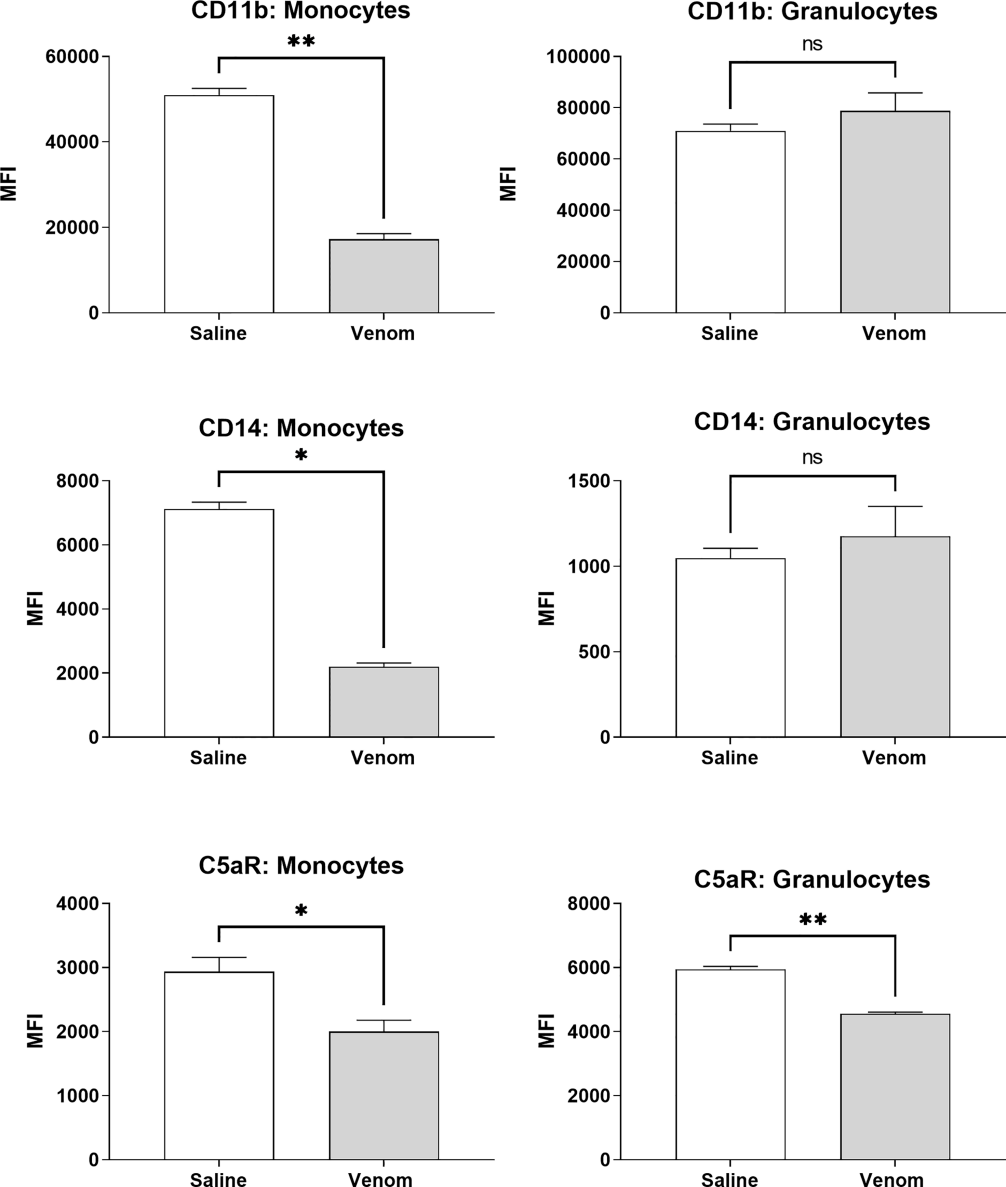
Expression of surface markers in monocytes and granulocytes after treatment with *B. jararaca* venom. Human blood samples containing GPRP (8 mg/mL) were treated with *B. jararaca* venom (50 μg/mL) or sterile saline solution (negative control) for 60 minutes at 37°C. After incubation, cells were analyzed for the expression of CD11b, CD14 and C5aR. The results are expressed as MFI ± SEM of duplicates from three independent experiments. ns, not significant; *p ≤ 0.05, **p ≤ 0.005.

**Figure 5** shows that *B. jararaca* venom induced a downregulation in the expression of CD11b, CD14 and C5aR in monocytes. C3aR, TLR2 and TLR4 showed no significant difference in expression when compared to the negative control (sterile saline solution) (**Supplementary Figure 1**). In granulocytes, the venom induced a reduction in C5aR expression, while CD11b, CD14, C3aR, TLR2 and TLR4 showed no significant difference compared to the negative control (sterile saline solution) (**Figure 5** and **Supplementary Figure 1**).

### Complement System Plays a Role in the Inflammation Induced by *B. jararaca* Venom in Human Whole Blood

Cp40 is a 14-amino acid nonimmunogenic cyclic peptide that binds to C3 and blocks its binding and cleavage by C3 convertase, inhibiting the generation of biologically active molecules. To assess whether Cp40 was capable of inhibiting the activation of the complement system stimulated by the venom of *B. jararaca* and, consequently, the generation of anaphylatoxins (C3a, C4a and C5a) and the terminal complex of the soluble complement (sTCC, SC5b-9), human blood samples were preincubated with Cp40 (20 μM) and then incubated with *B. jararaca v*enom (50.0 μg/mL) or sterile saline solution (negative control) for 60 minutes at 37°C.

**Figure 6:**
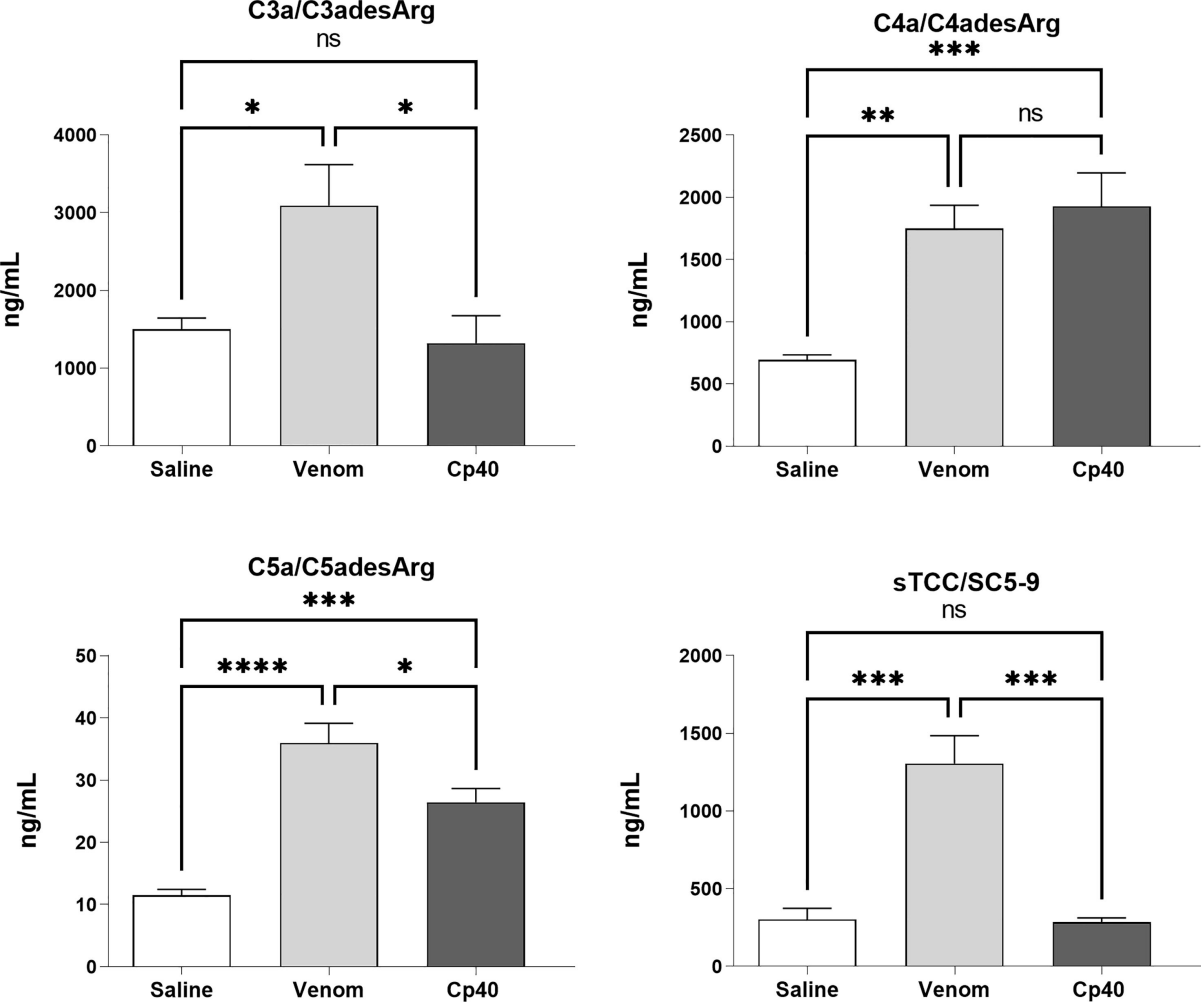
Production of anaphylatoxins in human blood stimulated with *B. jararaca* venom in the presence of Cp40. Human blood samples containing GPRP (8 mg/mL) were preincubated with cp40 inhibitor (20 μM) and then incubated with *B. jararaca* venom (50.0 μg/mL) or sterile saline solution for 60 minutes at 37°C. After plasma collection and dilution, the production of anaphylatoxins C3a/C3a-desArg (1:5000), C4a/C4a-desArg (1:5000) and C5a/C5a-desArg (1:1000) was evaluated by cytometric bead array (CBA). The results are presented as the mean ± SEM from three independent experiments. ns, not significant; *p ≤ 0.05, **p ≤ 0.005, ***p ≤ 0.0005, ****p < 0.0001.

**Figure 7:**
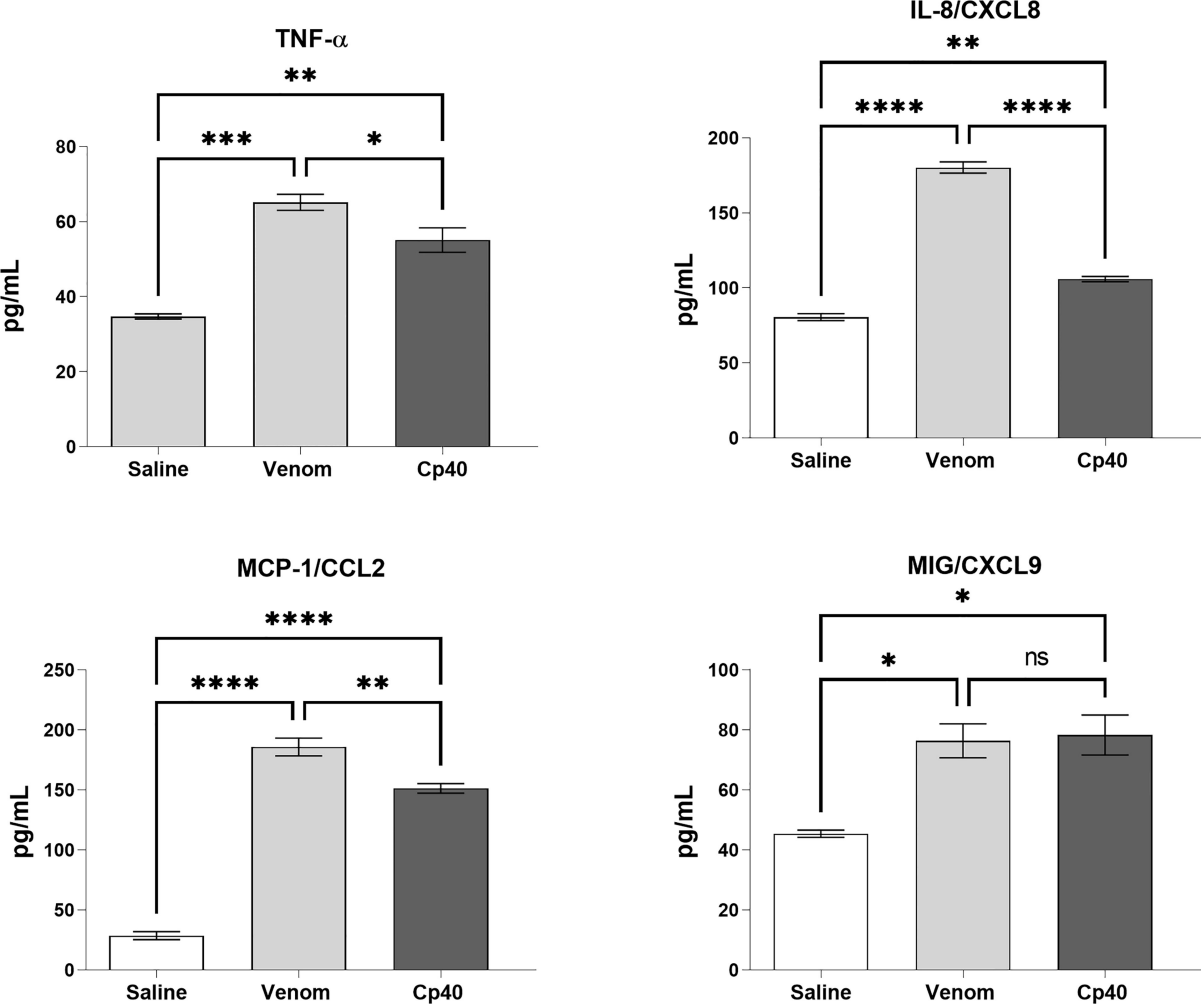
Levels of TNF-α and chemokines in human blood stimulated by *B. jararaca* venom in the presence of Cp40. Human blood samples containing GPRP (8 mg/mL) were preincubated with Cp40 inhibitor (20 μM) and then incubated with *B. jararaca* venom (50.0 μg/mL) or sterile saline solution for 60 minutes at 37°C. After plasma collection and dilution (1:2), the presence of TNF-α and the chemokines IL-8, MCP-1 and MIG was evaluated by the Cytometric Bead Array (CBA). The results are presented as the mean ± SEM from three independent experiments. ns, not significant; *p ≤ 0.05, **p ≤ 0.005, ***p ≤ 0.0005, ****p < 0.0001.

**Figure 8:**
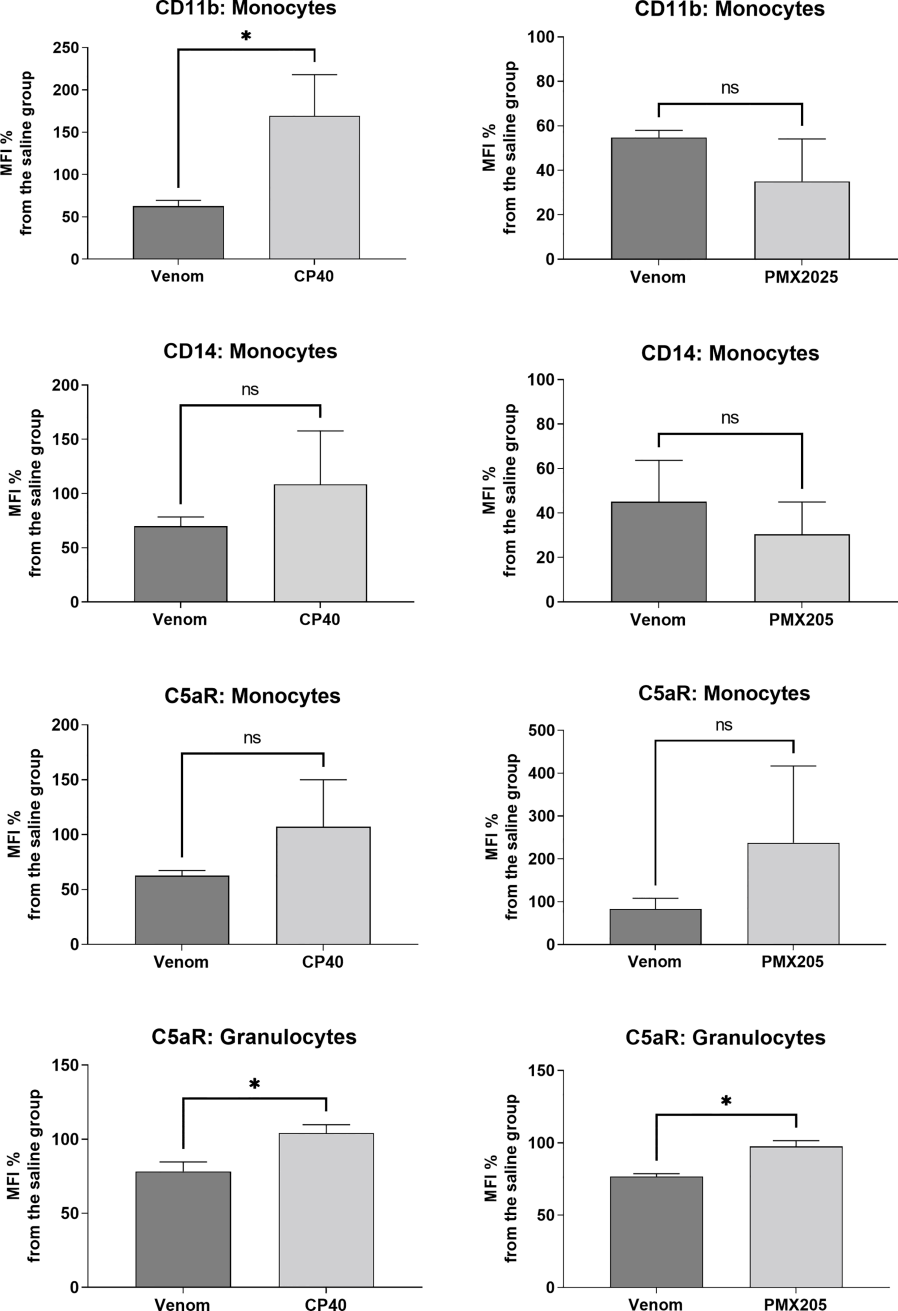
Expression of surface markers in monocytes and granulocytes after treatment with *B. jararaca* venom in the presence of C-inhibitors. Human blood samples containing GPRP (8 mg/mL) were pre incubated with Cp40 inhibitor (20 μM) or with PMX205 (10 μM) and then incubated with sterile saline (Cp40 vehicle), sterile saline + 5% glucose solution (PMX205 vehicle) or *B. jararaca* venom (50.0 μg/mL) for 60 minutes at 37°C. After incubation, cells were analyzed for the expression of CD11b, CD14 and C5aR. The results are expressed as MFI ± SEM of duplicates from three independent experiments. ns, not significant; *p ≤ 0.05.

**Figure 6** shows that Cp40 was able to significantly inhibit the generation of the anaphylatoxins C3a/C3a-desArg and C5a/C5a-desArg stimulated by the venom. However, the generation of C4a/C4a-desArg was not reduced and the levels of sTCC were also significantly reduced in the presence of Cp40 (**Figure 6**). The inhibition of the complement system by Cp40 significantly reduced the production of TNF-α, IL-8 and MCP-1 (but not MIG) induced by *Bothrops* venom in human blood (**Figure 7**). Moreover Cp40 reverted the reduction of CD11b expression in monocytes and C5aR in granulocytes (**Figure 8**).

**Figure 9:**
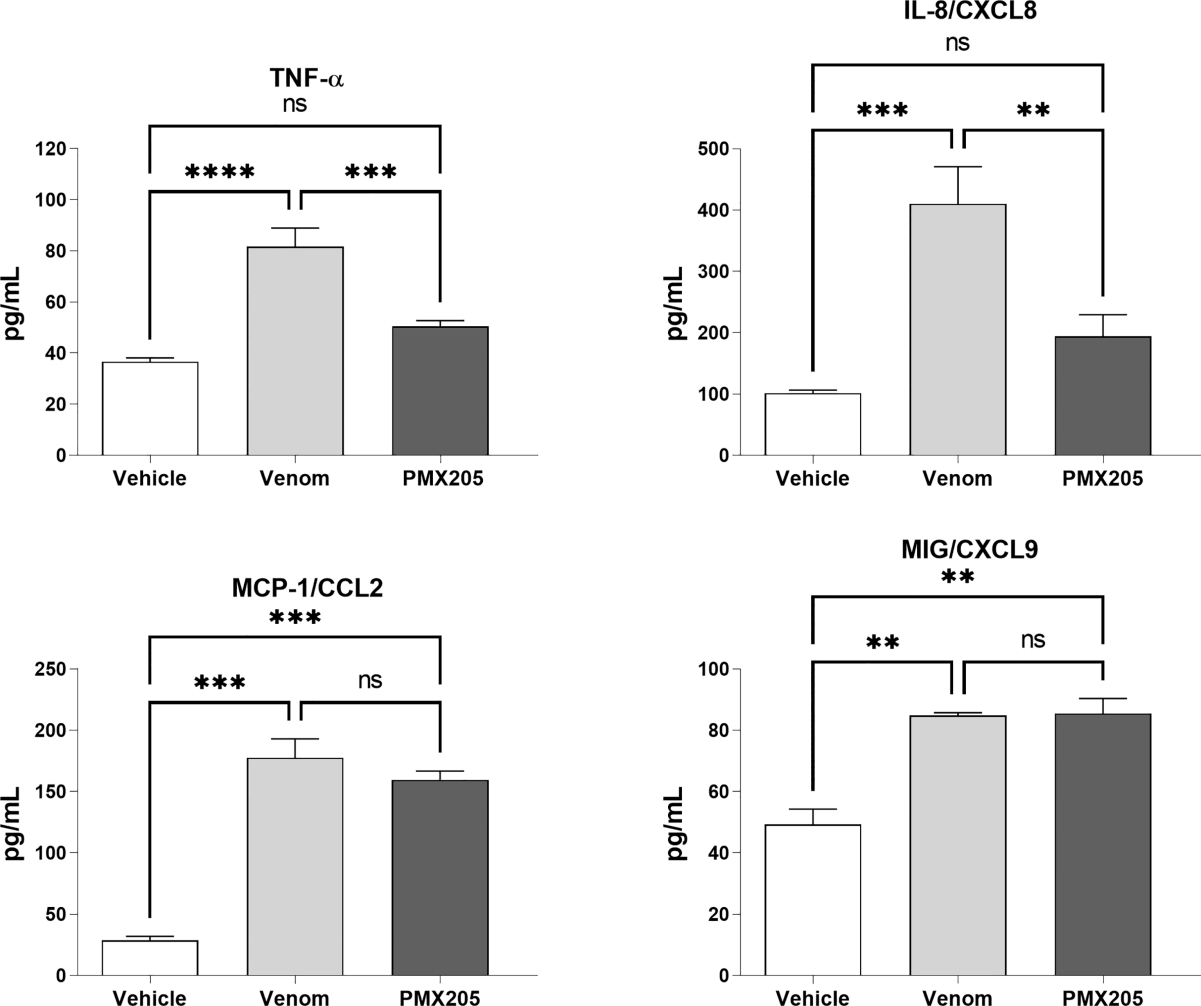
TNF-α and chemokine levels in human blood stimulated by *B. jararaca* venom in the presence of PMX205. Human blood samples containing GPRP (8 mg/mL) were preincubated with PMX205 (10 μM) and then incubated with *B. jararaca* venom (50.0 μg/mL) or sterile saline + 5% glucose solution for 60 minutes at 37°C. After plasma collection and dilution (1:2), the presence of TNF-α and chemokines was evaluated by the Cytometric Bead Array (CBA). The results are presented as the mean ± SD from three independent experiments. ns, not significant; **p ≤ 0.005, ***p ≤ 0.0005, ****p < 0.0001.

To evaluate the role of the C5a-C5aR1 axis in the production of proinflammatory mediators, human blood samples were preincubated with PMX205 (10 μM), a C5aR1 antagonist, and incubated with *B. jararaca* venom (50.0 μg/mL) or the negative control (sterile saline solution + 5% glucose). **Figure 8** shows that the inhibition of the C5a receptor by PMX205 promoted a reduction in the baseline levels of TNF-α and IL-8 in samples stimulated with the venom. However, PMX205 did not influence the production of the chemokines MCP-1 and MIG (**Figure 9**). PMX205 also positively modulated the C5aR expression on granulocytes (**Figure 8**).

## Discussion

The complement system plays an important role in pathogen immunosurveillance and tissue homeostasis. However, overactivation can lead to a cycle of inflammatory damage that exacerbates pathology.

Previous *in vitro* studies by our group have shown that venoms from snakes of the genus *Bothrops* are capable of activating the complement system in normal human serum with the production of large amounts of anaphylatoxins (22, 49). Delafontaine et al. (49) demonstrated that SVMPs are important for these effects because 1.10 Phe, an inhibitor of this class of proteases, drastically reduces C5a generation and sTCC formation in normal human serum incubated with the venom of *B. lanceolatus*. Furthermore, the C5a generated by direct cleavage of purified human C5 by proteases from this snake venom is highly functional, triggering activation of neutrophils characterized by an increase in the cytoplasmic levels of Ca^2+^. Furthermore, we detected that SVMPs reduced the inhibitory activity of the regulator of the classical and lectin pathways, C1-INH, *via* cleavage of this molecule. These results suggest that the activation of complement may play a role in the inflammatory process present in human envenomation by *Bothrops* snakes.

The human whole blood model, established by Mollnes et al. (43) and Brekke et al. (44, 45), was initially designed to study the role of complements in a bacterial sepsis model, and it also proved to be a useful experimental platform for the investigation and modulation of the systemic inflammation induced by animal venoms and toxins, as demonstrated by studies of our group with *Loxosceles* spider venom and its main toxin, sphingomyelinase D (50), and with a class P-I metalloprotease (C-SVMP) purified from the venom of *Bothrops pirajai* (51).

An adaptation by Johnson et al. (47) involving the substitution of lepirudin by GPRP, a peptide inhibiting the first steps of fibrin polymerization (52, 53), solved the issue that prevented studies from being conducted using the *ex vivo* human whole blood model with snake venom from the genus *Bothrops*. Bothropic venom exerts a procoagulant effect *in vitro*, which is determined by the presence of toxins with thrombin-like activity, factor II activators and factor X activators, in addition to other procoagulant toxins (54). The thrombin-like toxins cause the cleavage of the α and/or β chains of fibrinogen, which leads to rapid coagulation (55, 56). Thus, in the present study, GPRP was used as an anticoagulant, and we were able to overcome the thrombin-like toxin action and assess the proinflammatory action of crude *B. jararaca* snake venom in the *ex vivo* human whole blood model.

The data obtained here showed that the venom of *B. jararaca* induces activation of the complement system in human blood, as revealed by a significant production of anaphylatoxins (C3a/C3a-desArg, C4a/C4a-desArg and C5a/C5a-desArg) as well as of the soluble terminal complement complex (sTCC, SC5b-9) in a time- and dose-dependent manner (**Figures 1**, **2**). In general, the best time and concentration of incubation were 60 minutes and 50 μg/ml venom, respectively, which were selected for further experiments with complement inhibitors.

The anaphylatoxins C3a, C4a and C5a are biologically active fragments that MI are constantly released during the activation of the complement system. These mediators have a spectrum of proinflammatory activities that involve mast cell degranulation, increased vascular permeability, adhering molecule expression regulation, chemotaxis, leukocyte activation, oxygen-reactive species production, cytokines and chemokines, increased phagocytosis, and tissue regeneration (14, 20, 57). This wide set of effects promoted by anaphylatoxins is mediated by the expression of the seven-transmembrane receptors C3aR, C5aR1 (CD88), and C5aR2 (C5L2), and when activated in an exacerbated manner, these receptors can contribute to the development of several immunoinflammatory diseases (25, 58). In bothropic envenoming, the generation of leukocyte infiltrate and edema represent a characteristic of local inflammatory reactions (59–63), which may arise as a consequence of the abundant presence of anaphylatoxins, thereby highlighting the role of these mediators in venom-induced manifestations (49, 64, 65).

The complement terminal complex (TCC, sC5b-9) represents the final product of the cascade and is generated by the interaction of C5b with C6, C7, C8 and several molecules of C9. The membrane attack complex (sC5b-9 or MAC) is capable of generating pores in the cell membrane and favors lysis (17). In addition, the binding of this complex to endothelial cells provides stimuli capable of inducing the expression and release of molecules involved in leukocyte migration (66, 67).

Leukocyte migration in response to an inflammatory stimulus constitutes one of the first lines of defense assembled by innate immunity. This event is mediated by direct or indirect mechanisms. The latter involves the presence of cytokines and other chemotactic agents released by mast cells, macrophages and endothelial cells after antigen recognition (68). Here, we showed that the exposure of human blood to *B. jararaca* venom positively regulated the production of proinflammatory mediators, such as TNF-α and IL-8, at significant levels, especially in samples treated with the highest concentration of venom (50 μg/mL). This production was intensified, especially in the period of 120 minutes of incubation (**Figures 3**, **4**).

Tumor necrosis factor alpha (TNF-α) is synthesized predominantly by monocytes/activated macrophages and T lymphocytes as a 26 kDa protein, pro-TNF, which is associated with the plasma membrane, and when cleaved, the soluble form has an Mr of 17 kDa (69). It is a cytokine with potent action on various cell types and plays a critical role in the pathogenesis of several inflammatory diseases (70). Its interaction with endothelial cells, through one of two distinct receptors (TNFR1; CD120a, TNFR2; CD120b), mediates the release of chemokines (e.g., IL‐8, MCP‐1 and IP‐10) and the expression of different combinations of leukocyte-adhering molecules, including E-selectin, ICAM-1, VCAM-1 (71) and CD11b/CD18 (CR3; MAC-1) (72). Due to the ability of TNF-α to induce the expression of distinct classes of adhering molecules, this cytokine may be a collaborative factor for leukocyte infiltration in the presence of bothropic venom.

Interleukin-8 (IL-8; CXCL8) is a member of the chemokine family that acts through interaction with CXCR1 and CXCR2 receptors expressed on the surface of different cell types. IL-8 can be secreted by a wide variety of cells, including monocytes and T lymphocytes, after appropriate stimulation (73). In the acute inflammatory process, IL-8 plays an important role in phenomena related to neutrophil recruitment and activation, cytoskeleton reorganization, changes in intracellular Ca^2+^ levels, integrin activation, release of granular enzymes, and oxidative explosion (74, 75). The presence of increased serum levels of IL-8, IL-6, and TNF-α was observed in a clinical study with patients bitten by *B. asper* and *B. insularis* snakes (76).

Significant levels of MCP-1 and MIG chemokines were also observed. The generation of these chemokines was induced by the venom soon after 30 minutes of incubation with human blood and showed no marked decline in their levels until 120 minutes (**Figure 4**). The chemotactic protein of monocyte-1 (MCP-1), also known as CCL2, is part of a large family of structurally homologous proteins that support and regulate the movement of leukocytes from the blood to tissues. The recruitment of monocytes is highly regulated by this chemokine, which, at high concentrations, triggers oxidative explosion, generating reactive oxygen species (77, 78). MIG (Monokine induced by gamma interferon), also known as CXCL9, is a member of the CXC subfamily of inflammatory chemokines produced by dendritic cells, B lymphocytes and macrophages, which stimulates the recruitment of T lymphocytes to the sites of infection and/or injury by the CXCR3 receptor (79, 80).

In addition to the generation of proinflammatory mediators, the human whole blood model also allowed us to verify the leukocyte activation pattern by assessing the expression of cell surface molecules. A wide regulation of Toll-like receptor (TLR) signaling by the complement system was previously demonstrated in *in vivo* studies. Thus, TLR ligands, such as LPS (TLR4), zymosan (TLR2/6) and oligonucleotide CpG (TLR9), have been shown to induce complement-dependent production of TNF-α, IL-6 and IL-1β in mouse plasma (81, 82). A similar result was observed in mice treated simultaneously with TLR ligands and cobra venom factor (CVF), a potent complement activator isolated from *Naja naja* venom, with the production of elevated plasma levels of proinflammatory cytokines, indicating that the complement system can amplify the inflammatory response in association with Toll receptors (81, 82). Additionally, the involvement of anaphylatoxin receptors (C3aR and C5aR1/CD88) and CR3 (CD11b/CD18; MAC-1) in the regulation of TLR signaling activities has been shown (82–85).

In our assays, human blood samples were treated with *B. jararaca* venom, and the monocyte and granulocyte populations were analyzed for the expression of cell surface markers of interest*, i.e.*, TLRs 2 and 4; CD14; CD11b; C3aR and C5aR1. The data showed that *Bothrops* venom induced a significant reduction in the expression of CD11b, CD14 and C5aR1 in monocytes and of C5aR1 in granulocytes. Similarly, in an experimental model of sepsis induced in rodents, an important reduction in C5aR1 levels in neutrophils was demonstrated, and it was promoted by a markedly increased presence of C5a in the blood, which regulated the internalization of C5aR1 and whose intensity correlated with the lethality of the animals (86). Moreover, an additional mechanism for the reduction in the expression of C5aR1 is the cleavage and inactivation of C5aR1 by neutrophil serinoproteases (NSPs) (87).

The decrease in CD11b and CD14 expression in monocytes gate, induced by the venom, may suggest the presence of myeloid dendritic cell precursors in this population, that may have initiated a differentiation process into immature dendritic cells in response to the venom, despite the short incubation period. According to Patterson et al. (88), myeloid dendritic cell precursors purified from blood and cultured *in vitro* with GM-CSF and IL-4 rapidly differentiate into two maturational and phenotypically distinct populations, and the immature subtype is CD11b-/low and CD14- (88). The role of dendritic cells in the pathogenesis of snake envenomation is not clear, but it is known that snake toxins, such as crotoxin from *Crotalus durissus terrificus*, can activate dendritic cells and promote immunomodulation (89). Also, dendritic cells exert an essential role in the development of allergic reactions to bee and other highly allergenic venoms, due to their ability to direct the T helper immune responses (90). In our model, the possible differentiation of dendritic cells precursors by *B. jararaca* venom and its role in the envenomation remains to be further investigated.

To evaluate the participation of the complement system in pro-inflammatory events induced by the venom of *B. jararaca*, we conducted assays in the presence of Cp40 and PMX205 inhibitors. The use of Cp40, a highly specific inhibitor of the complement C3 component, led to a reduction in the baseline levels of C3a/C3a-desArg and sTCC in samples stimulated by the venom (**Figure 6**). It was also possible to observe a significant reduction in C5a/C5a-desArg levels but not in C4a/C4a-desArg levels (**Figure 6**). The absence of inhibition in the generation of C4a/C4a-desArg may be related to the direct action of metalloproteases present in bothropic venoms capable of cleaving components of the complement system (21, 22).

Previously, we investigated the inflammatory effects of a SVMP, named C-SVMP, isolated from *B. pitajai* venom in a human whole blood model (51). C-SVMP was able to activate the complement system and promote an increase in the expression of CD11b, CD14, C3aR, C5aR1, TLR2, and TLR4 markers in leukocytes. Inhibition of component C3 by compstatin significantly reduced complement activation induced by the toxin as well as CD11b, C3aR, and C5aR expression in leukocytes. C-SVMP was able to induce increased production of the cytokines IL-1β and IL-6 and the chemokines CXCL8/IL-8, CCL2/MCP-1, and CXCL9/MIG in the human whole blood model. The addition of compstatin, a C3 inhibitor, to the reactions caused a significant reduction in the production of IL-1β, CXCL8/IL-8, and CCL2/MCP-1 in cells treated with C-SVMP.

Here, we also showed that the complement system is involved in the synthesis of important inflammatory mediators detected in human blood treated with *B. jararaca* venom since inhibition of the central complement component C3/C3b *via* Cp40 resulted in a significant decrease in the production of TNF-α, IL-8 and MCP-1 (**Figure 7**). Moreover, the use of the C5aR1 antagonist PMX205 promoted a reduction in the baseline levels of TNF-α and IL-8 (**Figure 9**). Altogether, the data suggest that the C5a-CaR1 axis is essential for the production of TNF-α and IL-8 mediators induced by *Bothrops* venom in this *ex vivo* model. The fact that PMX205 was not able to reduce the production of MCP-1 and MIG (**Figure 8**) suggests that the production of these mediators is not influenced by the C5a-C5aR1 axis. CP40 was able to revert the CD11b and C5aR1 reduced expression induced by *B. jararaca* venom in monocytes, whereas PMX205 was able to improve the expression of C5aR1 in granulocytes.

Recently, we also evaluated the role of the complement system in the inflammatory events induced by *Naja annulifera* venom (33). This venom causes complement activation mediated by the action of SVMPs. The activation of the C5a-C5aR1 axis in the subcutaneous tissue of the animals injected with venom triggered the production of LTB_4_, PGE_2_ and TXB_2_, which were responsible for the edema. Additionally, the generation of venom-induced C5a led to the production of the chemokine CXCL1, along with an increase in MPO tissue levels. C5aR1 signaling in mice subjected to systemic envenomation was also responsible for leukocytosis, neutrophilia, monocytosis and acute lung injury, as demonstrated by the use of PMX205 in these assays.

In conclusion, the data presented here suggest that the activation of the complement system promoted by the crude venom of the snake *B. jararaca* in the human whole blood model contributes significantly to the inflammatory process. The control of several inflammatory parameters using Cp40, an inhibitor of component C3, and PMX205, a C5aR1 antagonist, suggests that the complement system may be a possible therapeutic target to control deleterious inflammatory reactions associated with envenomation by *Bothrops* snakes and other venomous animals in which the complement system is involved in the pathology of envenomation.

## Data Availability Statement

The original contributions presented in the study are included in the article/**Supplementary Material**. Further inquiries can be directed to the corresponding author.

## Ethics Statement

The studies involving human participants were reviewed and approved by the Human Research Ethics Committee from the SMS/SP (Secretaria Municipal da Saúde de São Paulo) (São Paulo, SP, Brazil) under the certificate number 3.813.859/CEP. The patients/participants provided their written informed consent to participate in this study.

## Author Contributions

Conceived and designed the experiments: TL and DT. Performed the experiments: TL and JG. Analyzed the data: TL, CS-B and DT. Contributed with reagents/materials/analysis tools: DT, TW, JL. Wrote the paper: TL, CS-B and DT. All authors read and approved the final manuscript.

## Funding

This work was supported by São Paulo Research Foundation (FAPESP) funding to the Center of Toxins, Immune Response and Cell Signaling (CeTICS) [grant 2013/07467-1]. DVT is a recipient of the CNPq Research Productivity Fellowship. The funding agencies had no influence on study design, data interpretation or formation of the manuscript.

## Conflict of Interest

JL is the founder of Amyndas Pharmaceuticals, which is developing complement inhibitors for therapeutic purposes; is the inventor of patents or patent applications that describe the use of complement inhibitors for therapeutic purposes, some of which are being developed by Amyndas Pharmaceuticals; is the inventor of the compstatin technology licensed to Apellis Pharmaceuticals (Cp05/POT-4/APL-1 and PEGylated derivatives such as APL-2/pegcetacoplan and APL-9). TW is an inventor on patents pertaining to complement inhibitors for inflammatory diseases. He has previously consulted to Alsonex Pty Ltd (who are developing PMX205), but holds not shares, stocks or other commercial interest in this company.

The remaining author declares that the research was conducted in the absence of any commercial or financial relationships that could be construed as a potential conflict of interest.

## Publisher’s Note

All claims expressed in this article are solely those of the authors and do not necessarily represent those of their affiliated organizations, or those of the publisher, the editors and the reviewers. Any product that may be evaluated in this article, or claim that may be made by its manufacturer, is not guaranteed or endorsed by the publisher.

## Supplementary Material

Supplementary Figure 1 Expression of surface markers in monocytes and granulocytes after treatment with *B. jararaca* venom. Human blood samples containing GPRP (8 mg/mL) were treated with *B*. *jararaca* venom (50 μg/mL) or sterile saline solution (negative control) for 60 minutes at 37°C. After incubation, cells were analyzed for the expression of C3aR, TLR2 and TLR4. The results are expressed as MFI ± SEM of duplicates from three independent experiments.
