# Supplementary figures and images for: Bothrops jararaca Snake Venom Inflammation Induced in Human Whole Blood: Role of the Complement System
Source: Front Immunol. 2022 Jun 2;13:885223. doi: 10.3389/fimmu.2022.885223 (PMC9201114; doi:10.3389/fimmu.2022.885223)

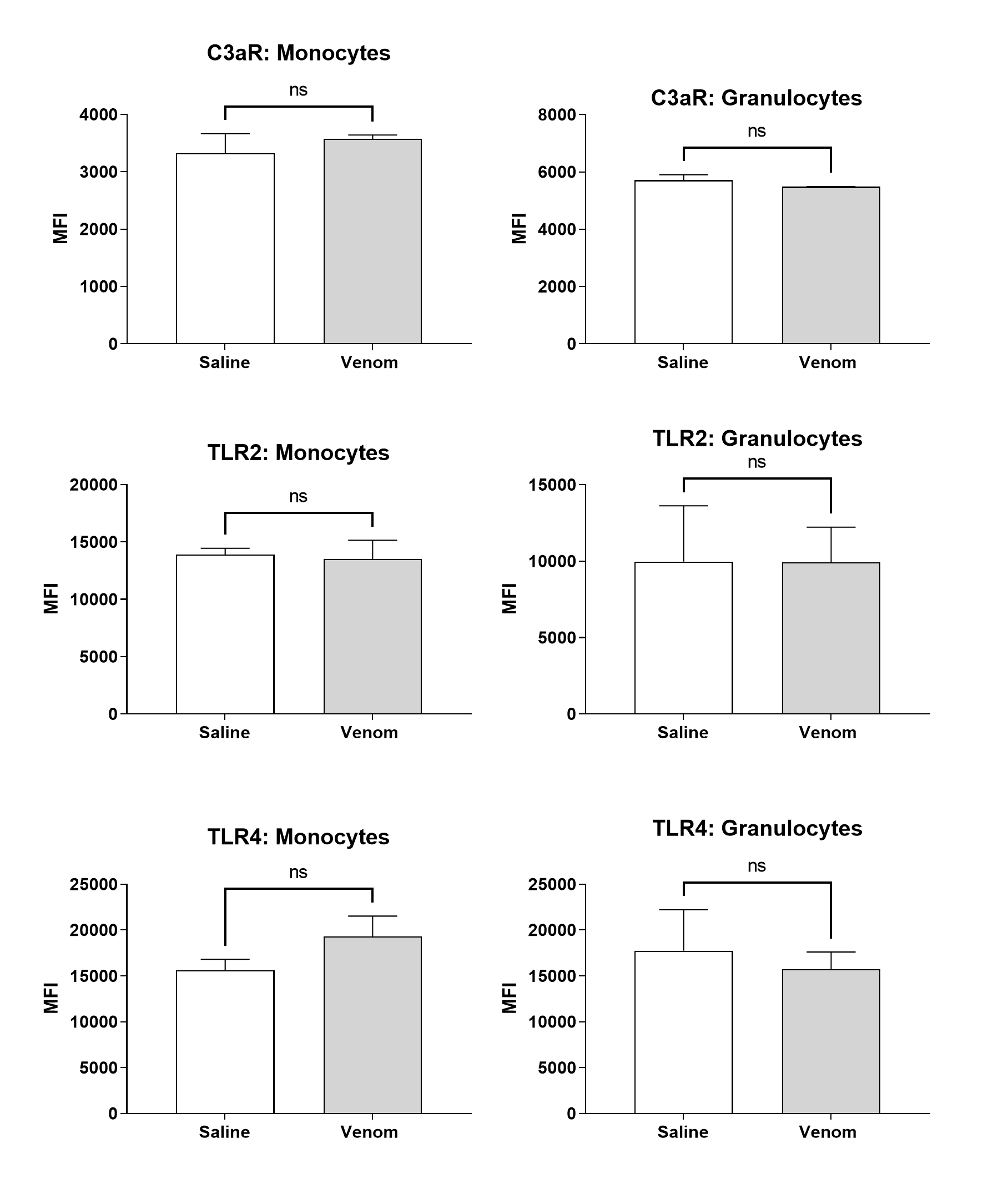

Supplement: Supplementary Figure 1 — Expression of surface markers in monocytes and granulocytes after treatment with B. jararaca venom. Human blood samples containing GPRP (8 mg/mL) were treated with B. jararaca venom (50 μg/mL) or sterile saline solution (negative control) for 60 minutes at 37°C. After incubation, cells were analyzed for the expression of C3aR, TLR2 and TLR4. The results are expressed as MFI ± SEM of duplicates from three independent experiments. [file Image_1.tif]
